# The effect of multidisciplinary extracorporeal membrane oxygenation team on clinical outcomes in patients with severe acute respiratory failure

**DOI:** 10.1186/s13613-018-0375-9

**Published:** 2018-02-27

**Authors:** Soo Jin Na, Chi Ryang Chung, Hee Jung Choi, Yang Hyun Cho, Kiick Sung, Jeong Hoon Yang, Gee Young Suh, Kyeongman Jeon

**Affiliations:** 1 Department of Critical Care Medicine, Samsung Medical Center, Sungkyunkwan University School of Medicine, 81 Irwon-ro, Gangnam-gu, Seoul, 06351 Republic of Korea; 2 Intensive Care Unit Nursing Department, Samsung Medical Center, Sungkyunkwan University School of Medicine, Seoul, Republic of Korea; 3 Department of Thoracic and Cardiovascular Surgery, Samsung Medical Center, Sungkyunkwan University School of Medicine, Seoul, Republic of Korea; 4 Division of Cardiology, Department of Medicine, Samsung Medical Center, Sungkyunkwan University School of Medicine, Seoul, Republic of Korea; 5 Division of Pulmonary and Critical Care Medicine, Department of Medicine, Samsung Medical Center, Sungkyunkwan University School of Medicine, Seoul, Republic of Korea

**Keywords:** Extracorporeal membrane oxygenation, Patient care team, Respiratory insufficiency, Critical care outcomes, Mortality

## Abstract

**Background:**

The Extracorporeal Life Support Organization (ELSO) has suggested that extracorporeal membrane oxygenation (ECMO) patients should be managed by a multidisciplinary team. However, there are limited data on the impact of ECMO team on the outcomes of patients with severe acute respiratory failure.

**Methods:**

All consecutive patients with severe acute respiratory failure who underwent ECMO for respiratory support from January 2012 through December 2016 were divided into the pre-ECMO team period (before January 2014, *n* = 70) and the post-ECMO team period (after January 2014, *n* = 46). Clinical characteristics and outcomes were compared between the two groups.

**Results:**

The mortality rates in the intensive care unit (72.9 vs. 50.0%, *P* = 0.012) and hospital (75.7 vs. 52.2%, *P* = 0.009) were significantly decreased in the post-ECMO team period compared to the pre-ECMO team period. The median duration of ECMO support was not different between the two periods. However, the proportion of patients successfully weaned off ECMO was higher in the post-ECMO team period (42.9 vs. 65.2%, *P* = 0.018). During ECMO support, the incidence of cannula problems (32.9 vs. 15.2%, *P* = 0.034) and cardiovascular events (88.6 vs. 65.2%, *P* = 0.002) was reduced after implementation of the ECMO team. The 1-year mortality was significantly different between the pre-ECMO team and post-ECMO team periods (37.8 vs. 14.3%, *P* = 0.005).

**Conclusion:**

After implementing a multidisciplinary ECMO team, survival rate in patients treated with ECMO for severe acute respiratory failure was significantly improved.

**Electronic supplementary material:**

The online version of this article (10.1186/s13613-018-0375-9) contains supplementary material, which is available to authorized users.

## Introduction

Recent studies showing the favorable results of extracorporeal membrane oxygenation (ECMO) have highlighted the role of ECMO in treating severe acute respiratory failure [1–4]. In addition, the number of patients receiving ECMO support in clinical practice is growing [4]. Despite the technical advances and generalization of the technique, ECMO is still a complex and costly treatment with potential adverse effects, and the clinical outcomes associated with its use are significantly different depending on the infrastructure of the providing center [5]. Therefore, the Extracorporeal Life Support Organization (ELSO) has published guidelines regarding the ideal institutional requirements for effective use of ECMO [6, 7]. In these guidelines, qualified ECMO physicians are referred to as one of the most important components of the successful implementation of ECMO, and their various responsibilities are emphasized, from initiation of ECMO to clinical follow-up [6, 7].

Some adjunctive therapies, such as use of neuromuscular blocking agents [8] and prone positioning [9], have been shown to reduce mortality in patients with severe acute respiratory failure, and these treatments before ECMO are also associated with outcomes seen after ECMO for respiratory failure [10, 11]. Therefore, decision making about the proper indications and timing of ECMO is a challenging problem for physicians who manage patients with severe acute respiratory failure. In addition, the medical management and nursing care of patients with severe respiratory failure receiving ECMO support are complex and can be challenging; therefore, the multidisciplinary ECMO team is recommended to be incorporated into ECMO program [7].

However, there are limited data on the impact of ECMO team on the outcomes of patients with severe acute respiratory failure. The objective of this study was to investigate the association between implementation of a multidisciplinary ECMO team and clinical outcomes in adult patients with severe respiratory failure receiving ECMO support.

## Methods

### Study design

**Fig. 1:**
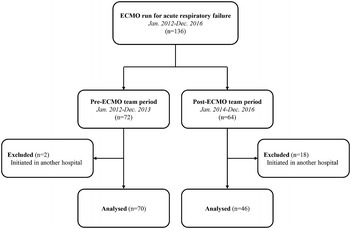
Patient distribution between the two periods

We conducted a retrospective cohort study between January 2012 and December 2016 at Samsung Medical Center (a 1979-bed tertiary referral hospital with tertiary-level intensive care units) in Seoul, South Korea. All patients 18 years of age or older for whom ECMO support was required for severe acute respiratory failure were enrolled in the study. A total of 136 ECMO runs in 133 patients were identified during this period. Twenty patients who were transported to our facility after initiation of ECMO in other hospitals were excluded because the decision regarding whether or not the patient was a suitable candidate for ECMO and initial management were not made by our ECMO team. The remaining 116 eligible ECMO runs were divided into the pre-ECMO team period (before January 2014, *n* = 70) and the post-ECMO team period (after January 2014, *n* = 46), according to the date of ECMO initiation (Fig. 1).

The institutional review board of the Samsung Medical Center approved this study and waived the requirement for informed consent because of the observational nature of the study. In addition, patients’ information was anonymized and deidentified prior to analysis.

### ECMO team and management of ECMO

In our institution, ECMO support has been available since 2004. In the first few years, veno-arterial ECMO was primarily used in patients with cardiac failure, with veno-venous ECMO used for less than five cases per year. The incidence of ECMO runs has gradually increased, with more than 100 cases currently performed annually. The application of veno-venous ECMO for severe respiratory failure has also grown and currently represents up to 20–30% of all ECMO runs.

Before 2014, there were standard criteria for indications and contraindications of ECMO (Additional file 1), but decisions about initiation and decannulation were mostly left to the physicians that oversaw patients. Cannula- or circuit-related issues were treated through elective consultation with cardiothoracic surgeons who had experience with ECMO. In 2014, our hospital adopted a multidisciplinary ECMO team consisting of cardiovascular surgeons, cardiologists, critical care physicians, and an ECMO specialist who is a cardiovascular perfusionist trained to manage the ECMO system and clinical needs of the patients on ECMO under supervision of ECMO-trained physicians. This ECMO team was responsible for every issue related to ECMO in the hospital. Protocols for indications and contraindications, management of patients and equipment, and weaning of patients from ECMO were revised. In addition, the ECMO team was charged with educating all medical personnel including bedside nurses caring for patients on ECMO at our institution. When a patient was deemed eligible for ECMO, the final decision to initiate ECMO was made by the treating intensivist and ECMO team, consisting of two more critical care physicians who are board certified in pulmonary and critical care medicine and cardiovascular surgeon, after a comprehensive assessment based on our protocol outlining the indications and contraindications. The primary cannulation strategy for adult respiratory ECMO was the veno-venous mode. The veno–veno-arterial mode was considered if the patient needed additional support due to hemodynamic failure. Cannulation was performed by the attending cardiothoracic surgeons using either the percutaneous method with the Seldinger technique or the surgical method at the bedside or in the operating room. Cannulation sites and cannula sizes were selected at the discretion of the cardiothoracic surgeons. Usually, a 20–28-Fr cannula was used for venous drainage via the common femoral vein, and a 14–18- or 20–24-Fr cannula was used for venous return via the internal jugular or the common femoral vein, respectively. The Prolonged Life Support System (Quadrox PLS, Maquet Inc., Rastatt, Germany) and the Capiox Emergency Bypass System (Capiox EBS; Terumo, Inc., Tokyo, Japan) were used. Pump blood flow and sweep gas flow rates were adjusted to maintain a target oxygen saturation and carbon dioxide removal rate. The mechanical ventilation (MV) strategy during ECMO was adapted from the study protocol of the CESAR trial [1], providing assisted pressure-controlled ventilation while limiting the peak inspiratory pressure to 25 cmH_2_O and applying positive end-expiratory airway pressure of 10 cmH_2_O, and respiratory rate of 10 breaths/min, on inspired oxygen fraction of 30%. Once the patients were stabilized and lightly sedated, spontaneous ventilation with pressure support mode was considered. In all patients, arterial catheterization was performed for continuous hemodynamic monitoring. Our ECMO team performed daily rounds and assessed the state of the ECMO circuit, development of ECMO-associated complications, and the possibility of weaning. An ECMO-trained physician provided 24-h on-call coverage, and an ECMO specialist participated in all intra-hospital transport of patients on ECMO. If a patient was considered ready to be weaned off ECMO support, decisions regarding decannulation were assessed through a protocolized weaning trial. Cannulae were removed at the bedside by cardiothoracic surgeons.

### Data collection and clinical outcomes

The clinical and laboratory data from patients who were treated with ECMO have been prospectively registered in the ECMO database of our hospital since 2012. For this study, these data were supplemented with a retrospective review of all hospital medical records. Demographic data, including age, sex, comorbidity, immune state, history of cardiac arrest, diagnosis, acute physiology and chronic health evaluation (APACHE) II score, and sequential organ failure assessment (SOFA) score, were recorded at admission to the intensive care unit (ICU). Presence of an artificial airway, use of MV, ventilator setting immediately before ECMO initiation, use of rescue and adjunctive treatment before ECMO, worst values from arterial blood gas and lactate tests within 6 h before ECMO initiation, respiratory extracorporeal membrane oxygenation survival prediction (RESP) score [11], predicting death for severe ARDS on VV-ECMO (PRESERVE) score [10], ECMO mode, and cannulation site were recorded on the first day of ECMO support.

The primary outcome in this study was in-hospital mortality. Secondary outcomes were ICU mortality, rate of weaning from ECMO, duration of ECMO support, adverse events during ECMO, rate of weaning from MV, duration of MV before weaning, ICU and hospital lengths of stay, and 1-year mortality after ECMO initiation. Adverse events during ECMO were defined as follows: cannula-related (vessel perforation with hemorrhage, arterial cannulation, malposition requiring repositioning, or accidental decannulation), other ECMO-related, hematological (gastrointestinal bleeding, cannula site bleeding, surgical site bleeding, plasma hemoglobin level > 50 mg/dL, or disseminated intravascular coagulation), neurological (brain death, seizure, cerebral infarction, or brain hemorrhage), cardiovascular (inotrope or vasopressor use, myocardial stunning, arrhythmia, cardiac tamponade, or cardiac arrest), pulmonary (pneumothorax or pulmonary hemorrhage), renal (serum creatinine level > 1.5 mg/dL or continuous renal replacement therapy), and infection (white blood cell count < 1500 × 10^3^ cells/mm^3^, culture-confirmed new infection, or ECMO-associated wound infection). Clinical outcomes were identified through medical record review. The Korean National Database, which uses citizen registration numbers, was used to obtain information about patient death at 1 year after ECMO initiation.

### Statistical analysis

To compare characteristics and clinical outcomes between the two periods, we analyzed categorical variables using *χ*^2^ tests or Fisher’s exact tests, when applicable, with data presented as numbers and percentages. Continuous variables were presented as medians with interquartile ranges, and Mann–Whitney *U* test was used for analysis. To adjust for potential confounding factors in the association between implementation of a multidisciplinary ECMO team and in-hospital mortality, multiple logistic regression analysis was used. Data are presented as adjusted odds ratios (ORs) with 95% confidence intervals (CI). Survival curves were constructed using the Kaplan–Meier method and compared with a log-rank test. For all analyses, a two-tailed test with a *P* value less than 0.05 was considered statistically significant. Statistical analyses were performed using SPSS 18.0 for Windows (Chicago, IL, USA).

## Results

### Baseline clinical characteristics

The baseline clinical characteristics of 116 patients separated according to whether their ECMO treatment period coincided with the implementation of the specialized ECMO team are shown in Table 1. Age, sex, and comorbidities were similar between the two periods. However, patients with airway disease and malignancy were more common in the pre-ECMO team period. Bacterial pneumonia was the most common pulmonary condition causing acute respiratory failure. The proportion of patients with bacterial pneumonia was higher in the pre-ECMO team period (45.7 vs. 26.1%; *P* = 0.033), while the proportion of patients with viral pneumonia was higher in the post-ECMO team period (2.9 vs. 17.4%, *P* = 0.014). The APACHE II (18 [15–25] vs. 25 [21–32], *P* < 0.001) and SOFA scores (6 [4–9] vs. 8 [6–14], *P* = 0.003) on the day of ICU admission were significantly higher in the post-ECMO team period compared with the pre-ECMO team period. However, the RESP and PRESERVE scores were similar between periods.

**Table 1:** Baseline patient characteristics

	Pre-ECMO team period (*n* = 70)	Post-ECMO team period (*n* = 46)	*P* value
Age (years)	61 (52–69)	60 (52–64)	0.672
Male	52 (74.3)	34 (73.9)	0.964
Body mass index (kg/m^2^)	23.1 (20.7–24.8)	23.8 (20.6–25.8)	0.163
Comorbidity			
Cardiovascular disease	7 (10.0)	5 (10.9)	>0.999
Chronic renal failure	6 (8.6)	3 (6.5)	>0.999
Asthma/COPD	12 (17.1)	2 (4.4)	0.039
Liver cirrhosis	3 (4.3)	3 (6.5)	0.680
Malignancy			0.052
Solid tumor	18 (25.7)^a^	6 (13.0)^b^	
Hematologic malignancy	11 (15.7)^c^	5 (10.9)^d^	
Immunocompromised state	23 (32.9)	17 (37.0)	0.650
Cardiac arrest before ECMO	8 (11.4)	10 (21.7)	0.134
Primary diagnosis			
Bacterial pneumonia	32 (45.7)	12 (26.1)	0.033
Viral pneumonia	2 (2.9)	8 (17.4)	0.014
Interstitial lung disease	15 (21.4)	5 (10.9)	0.141
Trauma/burn	2 (2.9)	4 (8.7)	0.212
Asphyxia	1 (1.4)	1 (2.2)	>0.999
Other^e^	18 (25.7)	16 (34.8)	0.294
Severity score on the first day in the ICU			
APACHE II	18 (15–25)	25 (21–32)	<0.001
SOFA	6 (4–9)	8 (6–14)	0.003
RESP score	1 (−1 to 2)	0 (−2 to 2)	0.171
PRESERVE score	5 (4–7)	6 (4–7)	0.664

Values are given as the median (interquartile range) or *n* (%)

*APACHE II* acute physiology and chronic health evaluation II, *COPD* chronic obstructive pulmonary disease, *ECMO* extracorporeal membrane oxygenation, *ICU* intensive care unit, *PRESERVE* predicting death for severe ARDS on VV-ECMO, *RESP* respiratory extracorporeal membrane oxygenation survival prediction, *SOFA* sequential organ failure assessment

^a^Meningioma (*n* = 1), malignant mesothelioma (*n* = 1), lung (*n* = 10), esophageal (*n* = 2), liver (*n* = 2), and colon cancer (*n* = 2)

^b^Acute myeloid leukemia (*n* = 3), acute lymphocytic leukemia (*n* = 1), chronic lymphocytic leukemia (*n* = 1), myelodysplastic syndrome (*n* = 4), and multiple myeloma (*n* = 2)

^c^Glioma (*n* = 1), lung cancer (*n* = 2), colon cancer (*n* = 2), and non-seminomatous germ cell tumor (*n* = 1)

^d^Acute myeloid leukemia (*n* = 1), multiple myeloma (*n* = 1), and lymphoma (*n* = 3)

^e^Other includes radiation therapy-induced pneumonitis, pulmonary tuberculosis, diffuse alveolar hemorrhage, pulmonary alveolar proteinosis, and airway occlusion by tumor mass or blood clot

### Medical management prior to ECMO

Treatment modalities for severe acute respiratory failure prior to ECMO initiation are presented in Table 2. The duration of MV prior to initiation of ECMO was similar in the pre- and post-ECMO team periods. Measurements performed during MV prior to ECMO, including positive end-expiratory pressure, peak inspiratory pressure, tidal volume per predicted body weight, and the worst values of arterial blood gases, were not different between the two periods. However, the partial pressure of oxygen in the arterial blood per fraction of inspired oxygen (PaO_2_/FiO_2_, PF) was significantly lower in the pre-ECMO team period (71.5 [56.0–96.5]) compared to the post-ECMO team period (91.8 [68.3–114.3]) (*P* = 0.033). As an adjunctive or rescue therapy for severe respiratory failure, steroids, neuromuscular blocking agents, prone positioning, and inhaled nitric oxide were used in 56.0, 52.6, 12.1, and 17.2% of overall cases, respectively. Patients in the pre-ECMO team period were more likely to receive steroids (65.7 vs. 41.3%, *P* = 0.010) and less likely to receive inhaled nitric oxide (11.4 vs. 26.1%, *P* = 0.041) or prone positioning (5.7 vs. 21.7%, *P* = 0.010) compared to the post-ECMO team period. Neuromuscular blocking agent usage was similar between the two periods (50.0 vs. 56.5%, *P* = 0.491).

**Table 2:** Medical management prior to extracorporeal membrane oxygenation

	Pre-ECMO team period (*n* = 70)	Post-ECMO team period (*n* = 46)	*P* value
Duration of MV before ECMO, days	2 (0–6)	2 (0–7)	0.510
Pre-ECMO ventilator settings^a^			
PaO_2_/FiO_2_	71.5 (56.0–96.5)	91.8 (68.3–114.3)	0.033
FiO2 (%)	100 (100–100)	100 (70–100)	0.009
PEEP (cmH_2_O)	10 (5–12)	10 (5–12)	0.239
Peak inspiratory pressure (cmH_2_O)	30 (24–34)	28 (23–31)	0.402
Minute volume (L)	9.1 (6.8–11.2)	9.5 (8.2–11.9)	0.420
VT/PBW (mL/kg)	6.8 (5.1–8.0)	6.8 (5.9–8.6)	0.710
Pre-ECMO treatment			
Steroid	46 (65.7)	19 (41.3)	0.010
Neuromuscular blocking agent	35 (50.0)	26 (56.5)	0.491
Inhaled nitric oxide	8 (11.4)	12 (26.1)	0.041
Prone position	4 (5.7)	10 (21.7)	0.010
Vasopressor infusion	44 (62.9)	29 (63.0)	0.984
Pre-ECMO blood gas			
pH	7.26 (7.13–7.38)	7.22 (7.09–7.35)	0.333
PaCO_2_ (mmHg)	58.1 (45.8–73.5)	57.8 (44.7–68.5)	0.699
PaO_2_ (mmHg)	61.4 (55.0–74.2)	64.2 (54.8–84.5)	0.726
HCO_3_ (mmol/L)	25.8 (21.8–30.2)	23.2 (19.2–28.2)	0.063
SaO_2_ (%)	89.4 (85.6–92.1)	89.4 (82.6–94.5)	0.890
Lactate before ECMO (mmol/L)	2.26 (1.53–4.80)	2.10 (1.60–5.11)	0.948

Values are given as the median (interquartile range) or *n* (%)

*ECMO* extracorporeal membrane oxygenation, *FiO*_*2*_ fraction of inspired oxygen, *MV* mechanical ventilation, *PaCO*_*2*_ partial pressure of carbon dioxide in arterial blood, *PaO*_*2*_ partial pressure of oxygen in arterial blood, *PBW* predicted body weight, *PEEP* positive end-expiratory pressure, *SaO*_*2*_ arterial oxygen saturations, *VT* tidal volume

^a^Data were available for 104 patients (66 patients in the pre-ECMO team period and 40 patients in the post-ECMO team period)

### ECMO management

Details on ECMO management are summarized in Table 3. Veno-venous ECMO was planned in all patients; in one case in the pre-ECMO team period, the cannula was unintentionally inserted into the common femoral artery, and ECMO support started in the veno-arterial mode. Another veno–veno-arterial mode was used in one case for additional hemodynamic support. Femoro-jugular configuration was present in 85.7% of cases, and femoro-femoral configuration was found in 11.4% of cases in the pre-ECMO team period, but the proportion of femoro-femoral cases increased to 28.3% in the post-ECMO team period.

**Table 3:** Management of extracorporeal membrane oxygenation

	Pre-ECMO team period (*n* = 70)	Post-ECMO team period (*n* = 46)	*P* value
Initial ECMO configuration			0.044
Femoro-jugular veno-venous	60 (85.7)	32 (69.6)	
Femoro-femoral veno-venous	8 (11.4)	13 (28.3)	
Femoro-femoral veno-arterial	2 (2.9)	0 (0.0)	
Mixed (veno–veno-arterial)	0 (0.0)	1 (2.2)	
Successful weaning off of ECMO	30 (42.9)	30 (65.2)	0.018
Duration of ECMO support (days)	10 (7–20)	11 (4–27)	0.674
Survivors	9 (5–16)	9 (4–23)	0.755
Non-survivors	15 (8–24)	19 (3–27)	0.754
Adverse events during ECMO			
ECMO-related complications			
Cannula	23 (32.9)	7 (15.2)	0.034
Malposition requiring repositioning	21	5	
Vessel perforation	1	0	
Arterial cannulation	1	0	
Accidental decannulation	0	2	
Other	11 (15.7)	11 (23.9)	0.271
Patient complications			
Hematological	20 (28.6)	10 (21.7)	0.411
Neurological	9 (12.9)	1 (2.2)	0.086
Cardiovascular^a^	62 (88.6)	30 (65.2)	0.002
Inotrope or vasopressor use	51	30	
Myocardial stunning	3	0	
Arrhythmia	19	5	
Cardiac tamponade	1	0	
Cardiac arrest	10	1	
Pulmonary	23 (32.9)	15 (32.6)	0.978
Renal	36 (51.4)	23 (50.0)	0.880
Infection	36 (51.4)	19 (41.3)	0.285

Values are given as the median (interquartile range) or *n* (%)

ECMO indicates extracorporeal membrane oxygenation

^a^One or more complications may be listed

Compared to the pre-ECMO team period (42.9%), the proportion of cases involving successful weaning from ECMO was significantly higher in the post-ECMO team period (65.2%) (*P* = 0.018). However, the median duration of ECMO support was not different between the two periods. Cardiovascular events were the most common complication in patients treated with ECMO, followed by pulmonary, infection-related, renal, and hematological complications. However, only the incidence of cardiovascular events was significantly different between the pre-ECMO team and post-ECMO team periods (88.6 vs. 65.2%, *P* = 0.002). There were cannula-related complications in 32.9% of cases in the pre-ECMO team period and in 15.2% of cases in the post-ECMO team period (*P* = 0.034). Other technical issues were not significantly different (Additional file 2).

### Clinical outcomes

**Fig. 2:**
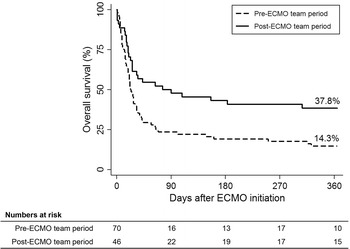
Overall survival at the 1-year follow-up. Cumulative survival 1 year after ECMO initiation according to the presence of an extracorporeal membrane oxygenation (ECMO) team

Patients were followed for a median of 573 (285–1231) days or until death after ECMO initiation. Although none of patients with malignancy had limitation of care decision at the time of ECMO initiation, do-not-resuscitate order was instituted in 9 (31.0%) out of 29 patients in pre-ECMO team period and 2 (18.2%) out of 11 patients in post-ECMO team period (*P* = 0.694). Overall, out of 116 ECMO patients, 77 (66.4%) deaths occurred during hospitalization. The ICU (72.9 vs. 50.0%, *P* = 0.012) and hospital (75.7 vs. 52.2%, *P* = 0.009) mortality rates were both significantly lower in the post-ECMO team period (Table 4). Also, the Kaplan–Meier survival curve showed a significant difference between the survival rates of the two periods during a 1-year follow-up period after ECMO initiation (*P* = 0.005) (Fig. 2). The rate of weaning from MV after ECMO was higher in the post-ECMO team period (56.5%) than in the pre-ECMO team period (30.0%) (*P* = 0.004) (Table 4). However, the median lengths of stay in the ICU and the hospital were not different between the two periods. The results of univariable and multivariable analyses with the logistic regression model are presented in Table 5. After adjusting for potential confounding factors, the post-ECMO team period was still significantly associated with lower in-hospital mortality (adjusted OR 0.11, 95% CI 0.03–0.46, *P* = 0.003). Other factors independently associated with in-hospital mortality were asthma/chronic obstructive pulmonary disease, malignancy, and RESP score (Table 5).

**Table 4:** Clinical outcomes

	Pre-ECMO team period (*n* = 70)	Post-ECMO team period (*n* = 46)	*P* value
Mortality			
Hospital	53 (75.7)	24 (52.2)	0.009
Intensive care unit	51 (72.9)	23 (50.0)	0.012
Length of stay (days)			
Hospital	36 (19–62)	39 (31–55)	0.528
Survivors	74 (32–118)	40 (34–72)	0.394
Non-survivors	32 (17–46)	37 (24–49)	0.725
Intensive care unit	28 (14–37)	25 (7–41)	0.633
Survivors	34 (17–63)	27 (7–42)	0.395
Non-survivors	28 (14–36)	24 (11–38)	0.796
Successful weaning off of mechanical ventilation	21 (30.0)	26 (56.5)	0.004
Duration of mechanical ventilation, days	18 (9–29)	15 (4–31)	0.253
Survivors	19 (8–28)	16 (4–39)	0.439
Non-survivors	18 (10–29)	14 (5–26)	0.246

Values are given as the median (interquartile range) or *n* (%)

ECMO indicates extracorporeal membrane oxygenation

**Table 5:** Univariable and multivariable analyses with logistic regression models for probability of in-hospital mortality

	Univariable			Multivariable		
	OR	95% CI	*P* value	OR	95% CI	*P* value
Post-ECMO team period	0.35	0.16–0.78	0.010	0.11	0.03–0.46	0.003
Asthma/COPD	7.72	0.97–61.36	0.053	10.76	1.17–99.04	0.036
Malignancy	2.76	1.12–6.78	0.027	3.97	1.21–13.01	0.023
Primary diagnosis^a^	–	–	–	–	–	–
Viral pneumonia	1.43	0.35–5.88	0.621	0.37	0.05–2.76	0.332
Others	1.30	0.33–5.12	0.706	0.76	0.12–4.97	0.773
APACHE II score	1.02	0.98–1.07	0.344	1.09	0.99–1.20	0.092
SOFA score	1.02	0.93–1.12	0.654	1.10	0.91–1.32	0.314
RESP score	0.85	0.73–0.99	0.034	0.77	0.62–0.96	0.020
PaO_2_/FiO_2_ prior to ECMO	1.00	0.99–1.01	0.776	1.02	1.00–1.04	0.058

*APACHE II* acute physiology and chronic health evaluation II, *COPD* chronic obstructive pulmonary disease, *ECMO* extracorporeal membrane oxygenation, *FiO*_*2*_ fraction of inspired oxygen, *MV* mechanical ventilation, *PaO*_*2*_ partial pressure of oxygen in arterial blood, *RESP* respiratory extracorporeal membrane oxygenation survival prediction, *SOFA* sequential organ failure assessment

^a^The reference group is bacterial pneumonia

## Discussion

Our study investigated the impact of a multidisciplinary ECMO team on clinical outcomes in patients who underwent ECMO for severe acute respiratory failure and found that implementation of this ECMO team was associated with significant reductions in ICU and hospital mortalities. The improvement in survival rates was maintained at the 1-year follow-up after ECMO initiation. Furthermore, the incidence of adverse events during ECMO support was reduced, and the successful weaning from ECMO and MV significantly increased after the ECMO team was implemented.

Our findings are consistent with the results of a previous study showing the beneficial impact on mortality rates of a program dedicated to ECMO in all adult and pediatric patients undergoing veno-arterial and veno-venous ECMO [12]. Several studies have demonstrated that the survival of patients with severe acute respiratory failure was significantly improved when treated with ECMO than when treated with conventional ventilation support and suggested that such patients be referred and transferred to an ECMO center [1, 13]. Nonetheless, long-term survival in this population has been more importantly associated with pre-morbid illnesses and functional ability at hospital discharge than with acute illness factors [14, 15]. However, our study found that ECMO management by a multidisciplinary team improved long-term outcomes in patients who underwent ECMO for severe acute respiratory failure. Therefore, our findings support the establishment of ECMO referral centers that include dedicated ECMO staffing in order to enhance the effective use of ECMO to improve long-term survival as well as to overcome acute illnesses in patients with severe acute respiratory failure [6, 7].

The beneficial effects associated with a specialized ECMO team in patients receiving ECMO might be related to multiple factors. First, patient selection for ECMO can be a possible explanation for these beneficial effects. ECMO initially emerged as a salvage therapy in patients with severe acute respiratory failure who cannot maintain adequate oxygenation or carbon dioxide removal despite MV; now, however, it is expanding its role beyond just being a salvage therapy, and some studies report that early initiation of ECMO is associated with lower mortality [16]. However, the disadvantages of ECMO might outweigh the advantages when implementing ECMO too early, given that complications, which can be serious and sometimes fatal, are possible throughout the entire course of ECMO support [4, 17]. Therefore, it is important to decide when and to whom we should apply ECMO for respiratory support. In this study, the proportion of patients with malignancy, which is usually considered to be a contraindication of ECMO, was higher than in previous ECMO studies. Although acute respiratory failure is one of the most common causes of ICU admission in patients with malignancies, the outcomes of ECMO in this population are disappointing [18], with only few cases reported to be successful [19, 20]. After the multidisciplinary ECMO team was implemented in our hospital, decisions about ECMO initiation were made by this ECMO team through comprehensive assessment with relevant consultants. As a result, the rate of survival to hospital discharge in patients with malignancy increased from 13.8 to 36.4% between the pre-ECMO team and post-ECMO team periods, respectively. The 1-year survival rate also increased from 3.4 to 18.2% after ECMO team implementation.

The duration and settings of MV and adjunctive therapies prior to ECMO are also known to be associated with differences in prognosis [10, 11, 21]. Although most parameters measured during ventilation were similar between the two periods in our study, the PF ratio was significantly lower in the post-ECMO team period. There was no difference in the duration of MV prior to ECMO initiation between the two periods, but it is possible that ECMO in the post-ECMO team period was started early in patients with less severe form of respiratory failure than in the pre-ECMO team period.

Next, the beneficial effects of the ECMO team can be explained by the dedication of the experienced and skilled ECMO physicians and staff members to ECMO. ELSO guidelines recommend that ECMO physicians should have sufficient experience and expertise in critical care and ECMO [6, 7]. Several previous studies revealed that a higher hospital-wide volume of ECMO cases was associated with lower mortality in patients who underwent ECMO [5, 22, 23]. Similarly, a specialized ECMO team will experience a greater volume of ECMO cases and be able to improve the skills associated with ECMO when they are responsible for ECMO throughout the entire center, rather than when managing only selected ECMO cases presented as elective consultations. The structure of an ECMO team could be similar to that of the high-intensity ICU staffing model, which is defined as mandatory intensivist consultation or the presence of a dedicated intensivist in the ICU [24]. The clinical benefit of the high-intensity staffing model over the low-intensity staffing model, which is defined as the absence of an intensivist or elective, rather than mandatory, intensivist consultation, in critically ill patients was already identified in several studies [25, 26].

Although this study provides new information on the impact of a multidisciplinary ECMO team on the clinical outcomes of adult patients with severe respiratory failure receiving ECMO support, our study has some limitations that should be considered. First, because it was conducted as a retrospective cohort study in a single center, there is a potential risk of confounding variables and selection bias. For example, the frequency of malignancy, which was considered to be a factor associated with poor outcomes, was significantly different between the two periods. Although it could also be considered as an effect of the ECMO team, however, the data regarding how many cancer patients had refused ECMO support by the team could not be extracted from the medical records during the study period. Second, the potential influence associated with the time difference between the two periods could not be excluded. Especially, growing experience with number of cases during the study period should be considered. Volume–outcome relationship in ECMO might stem from the beneficial effects of more experienced practitioners [27]. Recently, the ELSO registry has described improved outcomes in patients supported with ECMO over time, which might be attributed to the accumulation of experience [4].

In addition, the results from several studies that investigated the treatment of acute respiratory distress syndrome published during our study period might have influenced our practice, although the effects of these treatments on clinical outcomes in patients on ECMO remain unclear. Third, another research question was to identify changes in the selection of patients before and after implementation of the ECMO team. However, the data regarding how many patients with severe acute respiratory failure had been refused ECMO for respiratory support could not be extracted from the medical records during the study period before and after implementation of ECMO team. Further investigation with a larger patient population is needed to clarify the proper selection of patients for ECMO by the multidisciplinary team and the association of proper patient selection with clinical outcomes.

## Conclusion

After implementing a multidisciplinary ECMO team, short- and long-term survival rates were significantly improved in patients treated with ECMO for severe acute respiratory failure. Our findings support the recommendation that ECMO centers should have a specialized organization including ECMO staff members who are well qualified and have experience in ECMO in order to maximize the beneficial effects of this treatment in patients with acute respiratory failure.

## Additional files

**Additional file 1.** Criteria for indication and contraindication in pre- and post-ECMO team periods.

**Additional file 2.** Trends in complication rates during ECMO over the study period.

## Abbreviations

APACHE: acute physiology and chronic health evaluation
ECMO: extracorporeal membrane oxygenation
ELSO: Extracorporeal Life Support Organization
ICU: intensive care unit
MV: mechanical ventilation
PF: partial pressure of oxygen in the arterial blood per fraction of inspired oxygen (PaO_2_/FiO_2_)
SOFA: sequential organ failure assessment

## References

[1] Peek GJ, Mugford M, Tiruvoipati R, Wilson A, Allen E, Thalanany MM (2009). Efficacy and economic assessment of conventional ventilatory support versus extracorporeal membrane oxygenation for severe adult respiratory failure (CESAR): a multicentre randomised controlled trial. Lancet.

[2] Patroniti N, Zangrillo A, Pappalardo F, Peris A, Cianchi G, Braschi A (2011). The Italian ECMO network experience during the 2009 influenza A(H1N1) pandemic: preparation for severe respiratory emergency outbreaks. Intensive Care Med.

[3] Pham T, Combes A, Roze H, Chevret S, Mercat A, Roch A (2013). Extracorporeal membrane oxygenation for pandemic influenza A(H1N1)-induced acute respiratory distress syndrome: a cohort study and propensity-matched analysis. Am J Respir Crit Care Med.

[4] Thiagarajan RR, Barbaro RP, Rycus PT, McMullan DM, Conrad SA, Fortenberry JD (2017). Extracorporeal Life Support Organization Registry International Report 2016. ASAIO J.

[5] Barbaro RP, Odetola FO, Kidwell KM, Paden ML, Bartlett RH, Davis MM (2015). Association of hospital-level volume of extracorporeal membrane oxygenation cases and mortality. Analysis of the extracorporeal life support organization registry. Am J Respir Crit Care Med.

[6] ELSO Guidelines For ECMO Centers, Extracorporeal Life Support Organization. https://www.elso.org/resources/guidelines.asp. Assessed July 2017.

[7] Combes A, Brodie D, Bartlett R, Brochard L, Brower R, Conrad S (2014). Position paper for the organization of extracorporeal membrane oxygenation programs for acute respiratory failure in adult patients. Am J Respir Crit Care Med.

[8] Papazian L, Forel JM, Gacouin A, Penot-Ragon C, Perrin G, Loundou A (2010). Neuromuscular blockers in early acute respiratory distress syndrome. N Engl J Med.

[9] Guerin C, Reignier J, Richard JC, Beuret P, Gacouin A, Boulain T (2013). Prone positioning in severe acute respiratory distress syndrome. N Engl J Med.

[10] Schmidt M, Zogheib E, Roze H, Repesse X, Lebreton G, Luyt CE (2013). The PRESERVE mortality risk score and analysis of long-term outcomes after extracorporeal membrane oxygenation for severe acute respiratory distress syndrome. Intensive Care Med.

[11] Schmidt M, Bailey M, Sheldrake J, Hodgson C, Aubron C, Rycus PT (2014). Predicting survival after extracorporeal membrane oxygenation for severe acute respiratory failure. The Respiratory Extracorporeal Membrane Oxygenation Survival Prediction (RESP) score. Am J Respir Crit Care Med.

[12] Cotza M, Carboni G, Ballotta A, Kandil H, Isgro G, Carlucci C (2016). Modern ECMO: why an ECMO programme in a tertiary care hospital. Eur Heart J Suppl..

[13] Noah MA, Peek GJ, Finney SJ, Griffiths MJ, Harrison DA, Grieve R (2011). Referral to an extracorporeal membrane oxygenation center and mortality among patients with severe 2009 influenza A(H1N1). JAMA.

[14] Wang CY, Calfee CS, Paul DW, Janz DR, May AK, Zhuo H (2014). One-year mortality and predictors of death among hospital survivors of acute respiratory distress syndrome. Intensive Care Med.

[15] Enger TB, Philipp A, Lubnow M, Fischer M, Camboni D, Lunz D (2017). Long-term survival in adult patients with severe acute lung failure receiving veno-venous extracorporeal membrane oxygenation. Crit Care Med.

[16] Kanji HD, McCallum J, Norena M, Wong H, Griesdale DE, Reynolds S (2016). Early veno-venous extracorporeal membrane oxygenation is associated with lower mortality in patients who have severe hypoxemic respiratory failure: a retrospective multicenter cohort study. J Crit Care.

[17] Fan E, Gattinoni L, Combes A, Schmidt M, Peek G, Brodie D (2016). Venovenous extracorporeal membrane oxygenation for acute respiratory failure: a clinical review from an international group of experts. Intensive Care Med.

[18] Azoulay E, Mokart D, Pene F, Lambert J, Kouatchet A, Mayaux J (2013). Outcomes of critically ill patients with hematologic malignancies: prospective multicenter data from France and Belgium—a groupe de recherche respiratoire en reanimation onco-hematologique study. J Clin Oncol.

[19] Aboud A, Marx G, Sayer H, Gummert JF (2008). Successful treatment of an aggressive non-Hodgkin’s lymphoma associated with acute respiratory insufficiency using extracorporeal membrane oxygenation. Interact CardioVasc Thorac Surg.

[20] Liao WI, Tsai SH, Chiu SK (2013). Successful use of extracorporeal membrane oxygenation in a hematopoietic stem cell transplant patient with idiopathic pneumonia syndrome. Respir Care..

[21] Pranikoff T, Hirschl RB, Steimle CN, Anderson HL, Bartlett RH (1997). Mortality is directly related to the duration of mechanical ventilation before the initiation of extracorporeal life support for severe respiratory failure. Crit Care Med.

[22] Karamlou T, Vafaeezadeh M, Parrish AM, Cohen GA, Welke KF, Permut L (2013). Increased extracorporeal membrane oxygenation center case volume is associated with improved extracorporeal membrane oxygenation survival among pediatric patients. J Thorac Cardiovasc Surg.

[23] Freeman CL, Bennett TD, Casper TC, Larsen GY, Hubbard A, Wilkes J (2014). Pediatric and neonatal extracorporeal membrane oxygenation: does center volume impact mortality?*. Crit Care Med.

[24] Kim MM, Barnato AE, Angus DC, Fleisher LA, Kahn JM (2010). The effect of multidisciplinary care teams on intensive care unit mortality. Arch Intern Med.

[25] Pronovost PJ, Angus DC, Dorman T, Robinson KA, Dremsizov TT, Young TL (2002). Physician staffing patterns and clinical outcomes in critically ill patients: a systematic review. JAMA.

[26] Wilcox ME, Chong CA, Niven DJ, Rubenfeld GD, Rowan KM, Wunsch H (2013). Do intensivist staffing patterns influence hospital mortality following ICU admission? A systematic review and meta-analyses. Crit Care Med.

[27] Maclaren G, Pasquali SK, Dalton HJ (2014). Volume-outcome relationships in extracorporeal membrane oxygenation: is bigger really better?*. Crit Care Med.

