# Supplementary material for: The effect of multidisciplinary extracorporeal membrane oxygenation team on clinical outcomes in patients with severe acute respiratory failure
Source: Ann Intensive Care. 2018 Feb 27;8:31. doi: 10.1186/s13613-018-0375-9 (PMC5826909; doi:10.1186/s13613-018-0375-9)
Supplement: Supplementary file 1 — Additional file 1. Criteria for indication and contraindication in pre- and post-ECMO team periods. [file 13613_2018_375_MOESM1_ESM.docx]

**Additional file 1. Criteria for indication and contraindication in pre- and post-ECMO team periods**

| Pre-ECMO team period  (Jan. 2012 – Dec. 2013) | Post-ECMO team period  (Jan. 2014 – Dec. 2016) |
| --- | --- |
| 1. Patient selection | |
| Mostly determined at the discretion of the physicians that oversaw patients | Determined at the discretion of the treating intensivist and ECMO team, consisting of two more critical care physicians who are board certified in pulmonary and critical care medicine and cardiovascular surgeon |
| 2. Indications: Potentially reversible respiratory failure | |
| 1) Severe hypoxemia: PF ratio < 80 on FiO_2_ >90% and Murray LIS 3-4  2) Severe CO_2_ retention: PaCO_2_ > 80 mmHg or pH <7.2 on safe inflation pressure (Pplat ≤30 cmH_2_O)  3) Severe air leak syndromes | 1) Severe hypoxemia: PF ratio < 80 on FiO_2_ >90% and Murray LIS 3-4  2) Severe CO_2_ retention: PaCO_2_ > 80 mmHg or pH <7.2 on safe inflation pressure (Pplat ≤30 cmH_2_O)  3) Severe air leak syndromes  4) Bridge ECMO to lung transplantation (no evidence of existing organ failure) |
| 3. Contraindications | |
| 1) Duration of high pressure (P_plat_ > 30cmH_2_O) and high FiO_2_ (> 80%) ventilation >7 days  2) Intracranial hemorrhage and any other contraindication to systemic anticoagulation  3) Any contraindication to continue organ support | 1) Duration of high pressure (P_plat_ > 30cmH_2_O) and high FiO_2_ (> 80%) ventilation >7 days  2) Intracranial hemorrhage and any other contraindication to systemic anticoagulation  3) Any contraindication to continue organ support |
| 4. Patient and technical management during ECMO | |
| Patient's primary care physician is responsible for the patient's care during ECMO regardless of physician’s experience with ECMO. If cannula- or circuit-related issues occurred, it was treated through elective consultation with cardiothoracic surgeons who had experience with ECMO. | ECMO-trained physician managed patients on ECMO. The ECMO team daily assessed patients with ECMO and dealt with every clinical and technical issue related to ECMO in the hospital (24-hour on-call coverage). |
| 5. Physician and nursing staff education and training | |
| Manual on the management of ECMO patients existed, but regular systematic educational program was not performed. | ECMO team was charged with educating all medical personnel caring for patients on ECMO. Bedside nurses were regularly provided education and simulation-based training once a year. |
