# Supplementary material for: The effect of multidisciplinary extracorporeal membrane oxygenation team on clinical outcomes in patients with severe acute respiratory failure
Source: Ann Intensive Care. 2018 Feb 27;8:31. doi: 10.1186/s13613-018-0375-9 (PMC5826909; doi:10.1186/s13613-018-0375-9)
Supplement: Supplementary file 2 — Additional file 2. Trends in complication rates during ECMO over the study period. [file 13613_2018_375_MOESM2_ESM.docx]

**Additional file 2. Trends in complication rates during ECMO over the study period**

| ECMO-related complications | |
| --- | --- |
|  |  |
| Patient complications | |
|  |  |
|  |  |
|  |  |
